# Parental perceptions of barriers and facilitators to preventing child unintentional injuries within the home: a qualitative study

**DOI:** 10.1186/s12889-015-1547-2

**Published:** 2015-03-24

**Authors:** Joanne Ablewhite, Isabel Peel, Lisa McDaid, Adrian Hawkins, Trudy Goodenough, Toity Deave, Jane Stewart, Denise Kendrick

**Affiliations:** Division of Primary Care, University of Nottingham, Nottingham, NG7 2RD UK; Emergency Department, Nottingham University Hospitals NHS Trust, Nottingham, NG7 2UH UK; Clinical Research & Trials Unit, Norfolk and Norwich University Hospital NHS, Foundation Trust, Norwich, NR4 7UY UK; The Great North Children’s Hospital, Royal Victoria Infirmary, Newcastle Upon Tyne, NE1 4LP UK; Faculty of Health and Applied Sciences, University of the West of England, Bristol, BS16 1QY UK

**Keywords:** Child injury prevention, Qualitative, Child safety, Implications for injury prevention interventions

## Abstract

**Background:**

Childhood unintentional injury represents an important global health problem. Most of these injuries occur at home, and many are preventable. The main aim of this study was to identify key facilitators and barriers for parents in keeping their children safe from unintentional injury within their homes. A further aim was to develop an understanding of parents’ perceptions of what might help them to implement injury prevention activities.

**Methods:**

Semi-structured interviews were conducted with sixty-four parents with a child aged less than five years at parent’s homes. Interview data was transcribed verbatim, and thematic analysis was undertaken. This was a Multi-centre qualitative study conducted in four study centres in England (Nottingham, Bristol, Norwich and Newcastle).

**Results:**

Barriers to injury prevention included parents’ not anticipating injury risks nor the consequences of some risk-taking behaviours, a perception that some injuries were an inevitable part of child development, interrupted supervision due to distractions, maternal fatigue and the presence of older siblings, difficulties in adapting homes, unreliability and cost of safety equipment and provision of safety information later than needed in relation to child age and development. Facilitators for injury prevention included parental supervision and teaching children about injury risks. This included parents’ allowing children to learn about injury risks through controlled risk taking, using “safety rules” and supervising children to ensure that safety rules were adhered to. Adapting the home by installing safety equipment or removing hazards were also key facilitators. Some parents felt that learning about injury events through other parents’ experiences may help parents anticipate injury risks.

**Conclusions:**

There are a range of barriers to, and facilitators for parents undertaking injury prevention that would be addressable during the design of home safety interventions. Addressing these in future studies may increase the effectiveness of interventions.

## Background

Childhood unintentional injury continues to be an important public health issue both globally and within England [1-3]. In England, unintentional injuries occurring in or around the home are a leading cause of avoidable death and disability for children aged under five years [4]. Falls, thermal injuries and poisonings are the most common causes of emergency department (ED) attendances and hospital admissions [5]. In 2002, the latest year for which detailed emergency department (ED) data is available, unintentional injuries at home accounted for 416,806 ED attendances in children aged under 5 years; 58% of these were due to falls, poisoning or thermal injuries [6]. It is estimated that 90% of severe injuries in this age group are potentially preventable [7]. These attendances cost the NHS approximately £32 million [8], this does not include children treated by GP’s.

From birth to five years, rapid developmental changes occur which can result in children being susceptible to unintentional injury [9]. The patterns and types of injury are linked with the ages and stages of child development [10,11]. As children develop mobility, cognitive ability and receptive understanding parents need to anticipate injury risks and develop strategies to minimise risk.

A recent systematic review of quantitative literature identified a number of barriers and facilitators to the successful delivery of injury prevention interventions to parents [12]. Barriers related to socio-economic circumstances were identified and included issues such as low literacy levels and transient populations. Situations where families have limited financial resources for purchasing safety equipment and not having the appropriate tools to install safety equipment were also highlighted. Living in rented accommodation was identified as a barrier when parents were unable to install safety equipment in homes they did not own. Further barriers included trying to address too many safety issues and overly complex interventions. In addition language and cultural barriers were identified, for example some parents were suspicious or uncomfortable with home visits or having a stranger in their home. Parents’ resistance to changing their existing safety behaviours was also highlighted.

Facilitators to successful interventions included delivering interventions that had a clear and simple message, were simple for parents to implement and appropriately targeted to population groups. Providing safety equipment in addition to safety education and delivering interventions using child health professionals that were trusted or familiar figures, or had an established relationship with families were also found to have a facilitative effect.

A qualitative systematic review has also explored barriers and facilitators for effective interventions to reduce childhood unintentional home injuries. [13]. Barriers and facilitators were grouped into three categories; legal, policy or organisational; physical or environmental and individual.

The main barriers identified at the legal, policy or organisational level included weak legislation and a lack of appropriate information to parents. At the physical or environmental level barriers included living in rented accommodation and parents being unable to adapt the home as they would like, the cost of installing or maintaining safety devices and poor quality housing. Barriers identified at the individual level included parents perceiving unintentional injuries as inevitable, mistrust of officials and fear of being accused of abuse or neglect. A further barrier related to cultural differences with regard to practices, experiences and expectations [13].

The main facilitators identified at the legal, policy or organisational level included policy drivers and legislation, good communication between organisations and target audiences, involving local people and appropriate targeting of the population. At the physical or environmental level facilitators included stable and child friendly accommodation, having control over adapting the home to meet their child safety needs and having landlords that dealt with safety issues. Safety equipment use was also a facilitator and related to this was provision of appropriate and durable equipment, training in installation and maintenance of equipment. At the individual level the main facilitators were parental awareness of child injury risks, mothers safeguarding work, and teaching children about safety. Other facilitators included delivering safety information that was culturally sensitive, building trust and social connectedness rather than isolation [13]. A recent qualitative study highlighted that social networks are a facilitator of child safety awareness [14].

Understanding the barriers and facilitators to injury prevention experienced by parents in their day to day lives is crucial to the successful development and delivery of injury prevention interventions, strategy and policy. The study described in this paper is a multi-centre qualitative study undertaken as part of the National Institute for Health Research funded ‘Keeping Children Safe at Home (KCS) programme of research [15-18]. The aims of this study were two-fold: firstly, to explore parents’ perceptions of facilitators and barriers to keeping their children safe from unintentional injury within their home; and secondly, to understand parents’ perspectives of what might help them to implement injury prevention activities.

## Methods

### Recruitment and sampling

The aim was to recruit sixty-four parents of children aged less than 5 years, across four study centres (Nottingham, Bristol, Norwich and Newcastle). The sample was recruited from parents who had already taken part in multi-centre case–control studies [15-17] investigating risk and protective factors for childhood injuries. This included 48 (16 per centre) parents of cases from the case–control studies who had sought medical attention for their child following an unintentional fall, poisoning or scald within their home. It also included sixteen parents (across the four study centres) of controls in the case control studies who had not sought medical attention for an injury in their child.

Parents were recruited to the case–control studies face-to-face or by post. Those recruited face-to-face were provided with information during recruitment about three nested studies, of which this was one, and were asked if they wished to take part in any of these studies. Interviews were arranged with those agreeing to participate in this study. Participants recruited by post, who expressed an interest in any of the nested studies, were sent the study information leaflet and, at least 24 hours after anticipated receipt of the leaflet, were contacted by phone by a researcher to explain the study and arrange interviews with those agreeing to participate. Parents who participated in either of the other two nested studies were excluded from taking part in this study.

Prior to contacting or approaching parents, researchers checked that they would add to the maximum variation sampling frame [19]. The sampling frame was developed to ensure a range of participants were included in the study and comprised study centre, injury mechanism experienced by child (fall, poisoning, scald or control from the case- control studies) child age, child gender and the Index of Multiple Deprivation (IMD) 2010 [20] rank based on the postcode of the child’s home address. In order to complete the sampling, additional participants were recruited at two study centres (Bristol and Newcastle) because there were low numbers of parents of children with scald injuries in Norwich. Parents who took part in the study were given a £5 gift voucher, at the end of the interview, to thank them for their participation. Ethical approval was provided by the Nottingham 1 Ethics Committee (reference number: 09/H0407/14). Approval has been obtained from National Health Service research & development departments providing research governance to participating hospitals and Minor Injury Units.

### Data collection

Semi-structured interviews were used to elicit parents’ views on injury prevention in children within the home. An interview guide was developed based on findings from the systematic reviews of barriers and facilitators to injury prevention [12,13]. Four pilot interviews were undertaken; two in Nottingham and two in Bristol. Following the pilot, minor word changes and additional prompts were added. Data from the pilot interviews were not included in the analysis. The interview guide covered five topics: parental beliefs about injury prevention, strategies that can help to prevent injuries, parents or carers control over injury prevention actions, barriers to injury prevention actions, and facilitators for injury prevention actions. Interviews were digitally recorded, with the written consent of the participants. Interviews lasted between 30 and 60 minutes and were conducted in the parents’ home. Interviews were undertaken by a researcher from each study site. To maintain consistency, issues that arose throughout the course of the data collection process were discussed at regular teleconferences between researchers.

### Data analysis

Data was anonymised prior to transcription and transcribed verbatim. Initially, the data was read and re-read drawing out emerging themes and sub–themes; four transcripts were read by an independent research consultant, a lay research advisor and two researchers from different study sites to agree a coding structure. As the coding structure was applied to subsequent interview transcripts, other themes that emerged were discussed and agreed until a final set of themes was applied to all remaining interview transcripts. Data analysis was facilitated using the software Nvivo (version 9).

## Results

Sixty five parents participated in the study. One interview recording was inaudible and was excluded from the analysis. The characteristics of participants are shown in Table 1.

**Table 1:** **Participant characteristics**

	**Falls**	**Poisonings**	**Scalds**	**Controls**
**Child age**				
0-12 months	1	2	4	1
13-24 months	7	6	8	5
25-36 months	4	6	4	5
37-48 months	3	1	1	4
49-60 months	1	1	0	1
**Child gender**				
Male	9	10	8	9
Female	7	6	9	7
**Household composition**				
One parent in house	5	1	2	1
Both parents in house	10	15	13	15
One parent and other				
adults in house	0	1	2	1
Both parents and other				
adults in house	2	0	0	0
Not stated	0	0	1	0
**Number of children < 16 years in household**				
1 child	8	7	8	7
2 children	4	4	5	5
3 children	2	3	1	3
4 children	1	2	2	0
5 children	0	0	1	1
6 children	1	0	0	0
**Housing tenure**				
Live in rented house	7	4	11	2
Live in house owned or being purchased by family	8	12	6	14
Other	1	0	0	0
IMD rank*				
˂ median IMD rank	10	8	9	8
˃ median IMD rank	6	8	7	8
Median IMD rank = 16,241				

*IMD 2010 [21].

### Barriers to undertaking injury prevention within the home

Five main themes emerged relating to barriers to injury prevention: lack of anticipation of injury producing events by parents, the idea that there is little that can be done to prevent injuries, interrupted supervision, limitations with adapting the home, and the timing/targeting of safety information. The key themes are illustrated with anonymous quotes.

#### Parental anticipation of injury events

In terms of lack of parental anticipation of injury-producing events, three sub-themes emerged: parents anticipated injury producing events to some extent but this did not translate into preventive action; parents anticipated injury producing events but did not anticipate the severity of the resulting injury; and parents did not anticipate injury producing events because of their child’s age or developmental stage or because they thought they had already taken preventative action. Parents whose child had experienced a poisoning or scald tended to be more surprised by the injury event than parents whose child had experienced a fall.

*Not hugely surprised [that injury event occurred] surprised that she broke her collar bone. (Fall, female, age 3 years, ˂ IMD).*

*I was surprised because for one I’d never known him go on the worktop, like go to reach for anything on the worktop, I didn’t think he’d be able to reach, because of having two children before we know not to leave things on the edge of the worktop you know so it wasn’t and [B] said it wasn’t right on the edge it was kind of halfway back. But you know he was obviously determined and he was stretching as far as he possibly could. So yeah, it was a case of not realising how much he’d grown and – and erm, yeah – so I was surprised. (Scald, male, age 2 years, ˃ IMD).*

In the following example, the mother believed her medication was stored out of the child’s reach on a mantel piece. However, the child pulled down a necklace that was hanging from the mantel piece and the medication was dislodged and fell down into the child’s reach.

*I was extremely surprised. I am going to the kitchen just to pour her milk in the bottle which takes no longer than 3 minutes. By the time I got back she was sitting there under the mantel piece, round her all my stuff necklaces earrings rings everything plus my tablets. And there was one in the mouth - actually I could see it so I ran down I tried to take it out with my finger but if she knows that you want to do something she definitely wants to do the other. So she swallowed it as quickly as she could and there was nothing I could do about it. (Poisoning, female, age 2 years, ˂ IMD).*

#### ‘There is little that can be done to prevent injuries’

Some parents described how some injury-producing events were inevitable and therefore impossible to prevent. Parents of children who had experienced a fall injury described this more often than for poisonings and scalds.

*Kids are always gonna have accidents, you are not going to be able to prevent every one. (Fall, male, less than 1 year, ˂ IMD).*

*I don’t think anyone can stop anyone from doing anything to be fair…if someone thought that they could stop a child from having an accident then they are quite delusional cos then you’re going down the route of you can stop rape from happening, you can stop violence and all that stuff. You can’t. Some things are just going to happen (Fall male age 4 ˂ IMD).*

Some parents described a distinction between different types of injury, seeing injuries of lesser severity as inevitable and other injuries with more severe outcomes as preventable.

*Yeah obviously if it is gonna be potentially dangerous then prevent it or try to prevent it whereas falling over and having scrapes and tripping over things, you can’t help that generally (Fall male age 1 ˂ IMD).*

#### Interrupted supervision

A series of sub-themes emerged under this theme, including distractions, multi-tasking, maternal fatigue, the number of children in the household, the presence of older siblings and lone parenting. All parents described one or a combination of these themes. Trying to complete household tasks, supervise children, sometimes alone, and address multiple demands were examples of times when supervision might be interrupted.

*sometimes there’s only one of us here and things have to get done you know. You can’t sit down here with him watching him the whole time (Fall male age 2 ˂ IMD).*

*It’s difficult to try and get on with just daily tasks… You know like cooking and cleaning. It’s hard to do those kinds of things and watch [M] at the same time (Poisoning male age 4 ˃ IMD).*

Maternal fatigue was described as a barrier to injury prevention in terms lacking energy to provide direct and constant supervision.

*I mean I work full time, I work evenings, I am all over the place so I’ve always got so much to do, erm, so maybe like when you haven’t had enough sleep she is not a good sleeper at night so I mean the night before last we got about 2 and a half hours of sleep. So it’s easy to overlook something or forget something you have got a lot on your mind …and it’s just trying and I keep on top of everything so I think that is like my biggest worry or potentially when things can go wrong I mean that's how the accident happened (Fall female age 1 ˂ IMD)*

More than one child present was described by some parents as compromising their ability to supervise each child.

*And yeah it’s just a juggling act 3 kids you have always got to have eyes in the back of your head… He is little yeah and you forget that as well like when you have got a 5 year old and you got a baby, then 5 year old is still only a baby he is only young himself so you have got to be careful not to expect too much of them so erm coz he looks so much bigger as well than a baby you know (Poisoning male age 2 ˃ IMD)*

#### Difficulties with adapting the home

A series of sub-themes emerged under this theme, including restrictions relating to not owning the property, unreliability of safety equipment, not seeing the relevance of safety equipment, and the cost of safety equipment.

Living in rented accommodation was described as a barrier to injury prevention as parents were unable to improve the safety of their home in the way that they would like due to restrictions applied by the landlord.

*Especially with it not being mine because it’s a rented house. I can't put shelving across here. So I just sort of follow him around pretty much. I mean ideally I would put shelves up so I can move everything up a height and erm yeah put door catches on things you can't drill, erm the taps (bath taps) are quite hard yeah coz they are not mixer and it’s really like I am sure no matter how tight I tie them he can undo them (Fall male age 2 ˂ IMD).*

Mistrust or not seeing the relevance of safety equipment were identified as barriers and explained why parents often chose not to use such equipment in the home.

*we have got a baby lock on the cupboard with the cleaning stuff in down there, but even then I am not a 100% convinced that if I left her for too long that if she wasn’t supervised that she wouldn’t find some way and when we’ve had a fireguard or a stair gate they have just climbed up (Fall female age 3 ˃ IMD).*

#### Safety information: timing and targeting

Under the theme of timing of safety information, three sub-themes emerged including information arriving too late in relation to the ages and stages of child development, a lack of safety information or alternatively feeling bombarded by safety information.

*Because you get loads of support when the baby is really young and there’s loads of focus on breastfeeding erm – and that seemed to be the most information that I got when he was first born, it was all about that – and then as he gets older and he can walk you don’t get as much info. (Poisoning male age 4 ˃ IMD).*

*The trouble is I do think when you have got kids you get bombarded with so many leaflets from so many different places it could be about this that and everything that you tend to maybe either put them in a pile and not look at them anyway* (Fall male age 2 ˂ IMD).

### Facilitators

Five main themes emerged relating to facilitators to injury prevention. All parents described a combination of these strategies and the way that these strategies combined and/or altered with child age and development. The themes were predicting injury risk, parental supervision, teaching children about hazards and safety rules, adapting the home, and learning from other parents’ real life stories.

#### Predicting injury risk

In terms of predicting risk, some parents described how they try to anticipate injury risks and seek to minimise them either by supervision or by physically removing the hazard or child from the hazard.

*…the kind of the standard accidents if you know what I mean like the hot water in the kettles and knives and yeah they’re all kind of routinely mentioned in baby books and things like and the ones that you can foresee you know they are the predictable ones it’s the other ones with their imagination and creativity runs riot when you have problems. (Fall male age 3 ˃ IMD).*

#### Parental supervision

A series of sub-themes emerged under parental supervision. These varied from never leaving their child alone, described more often by parents whose child was aged less than two years, to knowing where the child was and listening for silence as a cue for parental intervention, mainly described by parents with a child older than two years.

*It’s just being on your guard at all times it don’t matter if you think like you’re cleaning stuff and that is in a safe place out of his reach coz they will still get to it. It’s knowing where they are. Listen out for the silence when it goes silence you know they are up to something. (Fall male age 2 ˂ IMD)*

*The stair gates are helpful of course they are helpful they are a tool that we do use and they can keep so you can you can go away or you can leave them unsupervised for a time but I think the most important thing is the supervision. (Control male age 2 ˃ IMD)*

#### Teaching children about hazards and safety rules

With regard to parental teaching strategies two sub-themes emerged; experiencing controlled risks, for example the parent letting the child feel the heat of the oven and outlining adverse consequences by explanation or through fear, for example explaining that household chemicals can make the child poorly. Having safety rules and sticking to them were also included in parents’ accounts of teaching.

*Because they learn right from wrong from an early age. They learn that no you can’t touch the kettle that it’s going to be hot and if you touch it, it is going to burn you. Same with the cooker you can’t reach and grab something off the cooker and things like that they need to still know what is right from wrong that if they touch something it is going to hurt them and if something is hot. (Control male age 3 ˂ IMD)*

*She knows under the kitchen cupboard was a naughty area and if she ever went to that cupboard I used to say ‘no that’s naughty’ and she wouldn’t even go in there… she was always very good at understanding not to touch so I have never had to kind of in a way make my home child friendly because she knew things were out of bounds… I always taught her no not to touch them and she was very quick at learning… (Poisoning female age 2 ˂ IMD).*

#### Adapting the home

Three sub-themes emerged with regard to adapting the home. These were minimising risks in rooms perceived as hazardous such as the kitchen and bathroom; placing items perceived as hazardous out of the child’s reach; and installing safety equipment.

*When they are younger obviously the safety equipment because you can’t teach them rules but you try as well but you need the equipment as well. (Control male child age 2 ˂ IMD)*

*No chemicals are kept in there (the kitchen), medicines are kept high up in an enclosed shelf. It’s not locked but you need two hands and to be an adult to get it out so it is not easy for the children. The children couldn’t reach it even with their steps.(Control male age 3 ˃ IMD)*

#### Learning from other parents’ ‘real life stories’

Learning from other parents’ ‘real life stories’ emerged as a theme as some parents described how this helped them to be aware of and anticipate injury risks.

*The iron I am really aware of because again that was an experience with someone that I knew had an iron dropped on himself when he was a baby and you know had brain damage from it so, so I am always really careful to think about that very much (Fall female age 2 ˂ IMD).*

### What might help parents to prevent unintentional injuries?

The parents who were interviewed were asked for suggestions about what might help them and other parents prevent unintentional child injuries in the home. Suggestions included information on safety for different ages and stages of development at appropriate times, learning from other parents, and knowing what to do if a child is injured.

Some parents said that they received lots of information when their child was a baby but less as the infant developed. Other parents described how they received information but that it was too late and they had already figured things out for themselves.

*Like for his age group, I mean you seem to get a lot of information for the younger ones and stuff like that but there’s nothing for, well not much on when he is 3 what can he reach, what can he do.(Control male age 3 ˃ IMD)*

Learning from other parents whose child has had an unintentional injury was suggested by some parents. This included learning about what happened and the kinds of risks to be aware of.

*actual case studies of what’s happened to people’s children so that they know that yes this can happen and it’s true life and to be aware (Poisoning female age 2 ˂ IMD).*

Support for parents so that they know what to do in the event of their child experiencing an injury was also suggested.

*After the accident, like when he did bump his head, I had no idea what I was meant to do like. I called the doctor, but I found after that from talking to my friends that nobody really knew what you were meant to do or what the signs were like. They gave me a leaflet after the hospital like if he throws up, but information like that would be really helpful to save unneeded visits or doing the wrong thing I think. (Fall male age 2 ˂ IMD).*

## Discussion

This study found a range of barriers that make it difficult, and some facilitators that help parents to prevent injuries to children within the home. Barriers included lack of anticipation of injury producing events, an acceptance that some injury events are inevitable, interrupted supervision, limitations to adapting the home and inappropriate timing / targeting of safety information in relation to the ages and stages of child development. Facilitators included a combination of parents predicting injury risks, parental supervision, teaching children about hazards and safety rules, adapting the home and learning from other parents’ ‘real life stories’. Many of these barriers are addressable within, and many facilitators could be exploited by injury prevention programmes.

Strengths of the study were that the sampling strategy ensured a cross-section of parent perspectives with parents living in more and less disadvantaged areas, children of sexes and varying ages, different injury mechanisms and injured and uninjured children. As a multi-centre study, it was also able to capture the perspectives of parents living in a range of localities. The use of a number of study centres also allowed flexibility, for example, it was possible to recruit additional participants at two study centres when there were low numbers of parents of children with thermal injuries at one study centre. A systematic approach was taken to the data analysis whereby multiple researchers were involved in the analytical process, helping to improve the rigor and quality of the findings [22]. This was facilitated with regular teleconferences and face-to-face meetings.

It is possible that parents who agreed to take part in the study had a particular interest in or were motivated by the aims of the study or child safety in general [23]. It is not appropriate to make generalisations from the findings of this study however, the maximum variation sampling, the large number of interviews conducted and the multi-centre nature of the study helped obtain a wide representation of views and experiences. These should be broadly transferrable to parents of young children living in similar situations.

Parental anticipation of injury risks is an important factor in preventing child home injury, where there is a lack of anticipation this has been highlighted as a barrier to injury prevention [13,24], this is supported by the findings of our study. Parental anticipation of child injury risk is complex and interwoven with a variety of factors [25]. Parents may anticipate injury risks but this may not translate into action due to a combination of factors. Such factors may include for example, maternal fatigue financial resources, multiple and competing demands for the parents’ attention. In addition, as has been previously found, parents may accept that some minor injuries are an inevitable aspect of early childhood [13,26,27]. With regard to ‘there is little that can be done to prevent injuries’ parents whose child had experienced a fall requiring attendance at ED described this more than parents whose child had experienced a poisoning or scald requiring attendance at ED. As has been previously found [28] it may be that some parents underestimate the likelihood of injuries perceived as ‘more serious’ and perceive ‘less serious’ injuries as more likely but an inevitable part of growing up. It may be that some parents do not anticipate the severity of injury outcomes from some activities, for example jumping from a bunk bed resulting in a broken collar bone.

Entwined with anticipation is parental supervision. Parental supervision encompasses a spectrum of activities to include watching, listening and awareness of where the child is and what the child is doing [25,29,30]. Supervision is an important factor for reducing injury risks [24,31-34] and parents in our study described supervision as a facilitator for reducing injury risk. However parents in our study also described barriers to injury prevention as times when supervision is interrupted as has been previously found [32,33,35]. It is also important to consider the factors that may affect supervision such as living in a home that is greater need of repair or a home that the parent does not own and are not free to child proof in the way that they might like. Such factors place greater pressures on parents to provide direct and constant supervision [36].

Consistent with other studies, we found living in a home the parent does not own can be a barrier to installing home safety equipment [12,13]. While some parents describe safety equipment as an aid to their injury prevention practices, as also found in other studies the perceived limitations of safety equipment can be a further barrier to its installation, maintenance and use [12,13] as can the financial cost of such equipment [12,13]. Social housing providers may be willing to engage with safety interventions as recently demonstrated for scald prevention [37,38], but enforcement through legislation, regulations or standards may also be required to address these barriers. Home safety interventions providing and fitting safety equipment may address some financial barriers and barriers relating to a lack of tools or skills to install and some equipment.

There is some evidence to suggest some parents prefer finding out and learning about safety through other parents rather than by talking with professionals [39]. Our study also found that parents may find learning from other parent’s experiences of injuries useful for developing anticipatory knowledge and planning preventative strategies [14,39].

Different injury risks are associated with different ages and stages of child development [10,11] and require different anticipatory and supervisory practices. Peer programmes, where appropriately trained parents provide home safety advice to parents, have demonstrated reductions in injury risk [40]. Social networks and appropriately trained mothers have also been suggested as methods of communicating safety messages to parents [14,39]. Our findings suggest providing safety information appropriate to child age and development, including through the use of “real life” stories from parents of injured children, may provide a way forward for delivering interventions.

### Implications for research and practice

Removing barriers to and enhancing facilitators for injury prevention during the development of home safety interventions has the potential to increase the effectiveness of interventions. It is important that future intervention studies report and explore barriers and facilitators to injury prevention to help understand why interventions are, or are not effective. Explanations regarding the implementation and effectiveness of interventions need to include the broader context in which parent’s injury prevention decision making and behaviours occur.

## Conclusions

There are a range of barriers to, and facilitators for parents undertaking injury prevention that would be addressable during the design of home safety interventions. Addressing these in future studies may increase the effectiveness of interventions.

## References

[1] Peden M, Oyegbite K, Ozanne-Smith J, Hyder A, Branche C, Rahman A (2008). World Report on Child Injury Prevention.

[2] Audit Commission (2007). Better safe than sorry. Preventing unintentional injury to children. National Report (Health).

[3] ONS. Table 2 Deaths Registration Summary Statistics. England and Wales 2011. 2011.

[4] Public Health England (2014). Reducing unintentional injuries in and around the home among children under five years.

[5] Hospital Episode Statistics, Admitted Patient Care, England 2012–13: External causes. Available at: http://www.hscic.gov.uk/catalogue/PUB12566/hosp-epis-stat-admi-ext-caus-2012-13-tab.xlsx. Accessed February 27, 2015

[6] RoSPA. HASS and LASS Home & Leisure Accident Surveillance System Annaul Report 2002 In*.* Edited by Dept of Trade and Industry: http://www.hassandlass.org.uk/reports/2002data.pdf; 2002.

[7] Joffe AR, Lalani A (2006). Injury admissions to pediatric intensive care are predictable and preventable: a call to action. J Intensive Care Med.

[8] Department of Health (2013). NHS Reference costs 2012–13.

[9] Sethi D, Towner E, Vincenten J (2008). European Report on Child Injury Prevention.

[10] Pickett W, Streight S, Simpson K, Brison RJ (2003). Injuries experienced by infant children: a population-based epidemiological analysis. Pediatrics.

[11] Agran PF, Anderson C, Winn D, Trent RB, Walton-Haynes L, Thayer S (2003). Rates of pediatric injuries by 3-month intervals for children 0 to 3 years of age. Pediatrics.

[12] Ingram JC, Deave T, Towner E, Errington G, Kay B, Kendrick D (2012). Identifying facilitators and barriers for home injury prevention interventions for pre-school children: a systematic review of the quantitative literature. Health Educ Res.

[13] Smithson J, Garside R, Pearson M (2011). Barriers to, and facilitators of, the prevention of unintentional injury in children in the home: a systematic review and synthesis of qualitative research. Inj Prev.

[14] Khanom A, Hill RA, Brophy S, Morgan K, Rapport F, Lyons R (2013). Mothers’ perspectives on the delivery of childhood injury messages: a qualitative study from the growing up in Wales, environments for healthy living study (EHL). BMC Public Health.

[15] Kendrick D, Maula A, Stewart J, Clacy R, Coffey F, Cooper N (2012). Keeping children safe at home: protocol for three matched case–control studies of modifiable risk factors for falls. Inj Prev.

[16] Wynn P, Stewart J, Kumar A, Clacy R, Coffey F, Cooper N (2014). Keeping children safe at home: protocol for a case–control study of modifiable risk factors for scalds. Inj Prev.

[17] Majsak-Newman G, Benford P, Ablewhite J, Clacy R, Coffey F, Cooper N (2014). Keeping children safe at home: protocol for a matched case–control study of modifiable risk factors for poisoning. Inj Prev.

[18] Keeping children safe programme. http://www.nottingham.ac.uk/research/groups/injuryresearch/projects/kcs/index.aspx [accessed 30/09/2014]

[19] Patton M (1990). Qualitative evaluation and research methods.

[20] English Indices of Deprivation 2010. Available at https://www.gov.uk/government/statistics/english-indices-of-deprivation-2010. Accessed February 27, 2015.

[21] Government CaL. The English Indices of Deprivation. In*.*: Direct Gov; 2010.

[22] Murphy E, Dingwall R, Greatbatch D, Parker S, Watson P (1998). Qualitative research methods in health technology assessment: a review of the literature. Health Technol Assess.

[23] Bowling A (2002). Research methods in health.

[24] Garling A, Garling T (1995). Mothers’ anticipation and prevention of unintentional injury to young children in the home. J Pediatr Psychiatry.

[25] McMullin J, Dao A (2014). Watching as an ordinary effect: care and mothers’ preemption of injury in child supervision. Subjectivity.

[26] Whitehead E, Owens D (2012). Parental perceptions of unintentional injury risks to children. Int J Health Promot Educ.

[27] Evans SA, Kohli HS (1997). Socioeconomic status and the prevention of child home injuries: a survey of parents of preschool children. Inj Prev.

[28] Glik D, Kronenfeld J, Jackson K (1991). Predictors of risk perceptions of childhood injury among parents of preschoolers. Health Educ Behav.

[29] Schwebel DC, Kendrick D (2009). Caregiver supervision and injury risk for young children: time to re-examine the issue. Inj Prev.

[30] Morrongiello BA (2005). Caregiver supervision and child-injury risk: I. Issues in defining and measuring supervision; II. Findings and directions for future research. J Pediatr Psychol.

[31] Sparks G, Craven MA, Worth C (1994). Understanding differences between high and low childhood accident rate areas: the importance of qualitative data. J Public Health Med.

[32] Roberts H, Smith SJ, Bryce C (1995). Children at risk? Safety as a social value.

[33] Ginnelly L, Sculpher M, Bojke C, Roberts I, Wade A, Diguiseppi C (2005). Determining the cost effectiveness of a smoke alarm give-away program using data from a randomized controlled trial. Eur J Pub Health.

[34] Kendrick D, Mulvaney C, Ye L, Stevens T, Mytton J, Stewart-Brown S. Parenting interventions for the prevention of unintentional injuries in childhood. Cochrane Database Syst Rev. 2013. 10.1002/14651858.CD006020.pub3 PMC8908963 23543542

[35] Boles RE, Roberts MC (2008). Supervising children during parental distractions. J Pediatr Psychol.

[36] Ablewhite J, Kendrick D, Watson M, Shaw I (2014). Maternal perceptions of supervision in pre-school-aged children: a qualitative approach to understanding differences between families living in affluent and disadvantaged areas. Prim Health Care Res Dev.

[37] Kendrick D, Stewart J, Smith S, Coupland C, Hopkins N, Groom L (2011). Randomised controlled trial of thermostatic mixer valves in reducing bath hot tap water temperature in families with young children in social housing. Arch Dis Child.

[38] Edwards P, Durand M, Hollister M, Green J, Lutchmun S, Kessel A (2011). Scald risk in social housing can be reduced through thermostatic control system without increasing Legionella risk: a cluster randomised trial. Arch Dis Child.

[39] Ablewhite J, Kendrick D, Watson MC, Shaw I: The Other Side of the Story - Maternal perceptions of safety advice and information. Child Health Care Dev. 2014. In Press. 10.1111/cch.12224 PMC4964917 25605523

[40] Barlow J, Johnston I, Kendrick D, Polnay L, Stewart-Brown S: Individual and group-based parenting programmes for the treatment of physical child abuse and neglect. Cochrane Database Syst Rev. 2006;Issue 3. Art. No.: CD005463. DOI: 005410.001002/14651858.CD14005463.pub14651852. 10.1002/14651858.CD005463.pub2 PMC13199909 16856097

